# Canine parvovirus type 2c identified from an outbreak of severe gastroenteritis in a litter in Sweden

**DOI:** 10.1186/1751-0147-55-64

**Published:** 2013-09-10

**Authors:** David Sutton, Carina Vinberg, Agneta Gustafsson, Jacqueline Pearce, Neil Greenwood

**Affiliations:** 1 MSD Animal Health, Walton Manor, Walton, Milton Keynes, MK7 7AJ, UK; 2 Helsingborg Animal Hospital, Box 220 97, Helsingborg, SE-250 23, Sweden; 3 MSD Animal Health, Box 7121, Sollentuna, SE-192 07, Sweden

**Keywords:** Dog, Haemorrhagic gastroenteritis, Vaccination, CPV2c, Sweden

## Abstract

A litter of recently-vaccinated puppies in Sweden experienced signs of severe haemorrhagic gastroenteritis. Canine parvovirus (CPV) was suspected as the cause of this outbreak on the basis of the clinical signs and the presence of parvoviral antigen in the faeces from one of the affected pups - confirmed using a commercial in-clinic faecal antigen ELISA test kit. A concern was raised about whether the vaccine (which contained a live, attenuated strain of CPV) could have caused the disease and so further faecal samples from the affected pups were submitted for laboratory virus isolation and identification.

On cell culture, two out of four faecal samples were found to be virus-positive. This was confirmed as being canine parvovirus by immuno-staining with CPV specific monoclonal antibody. The virus was then tested using a series of PCR probes designed to confirm the identity of CPV and to distinguish the unique vaccine strain from field virus. This confirmed that the virus was indeed CPV but that it was not vaccine strain. The virus was then typed by sequencing the 426 amino acid region of the capsid gene which revealed this to be a type 2c virus.

Since its emergence in the late 1970s, canine parvovirus 2 (CPV2) has spread worldwide and is recognised as an important canine pathogen in all countries. The original CPV2 rapidly evolved into two antigenic variants, CPV2a and CPV2b, which progressively replaced the original CPV2. More recently a new antigenic variant, CPV2c, has appeared. To date this variant has been identified in many countries worldwide but there have been no reports yet of its presence in any Scandinavian countries. This case report therefore represents the first published evidence of the involvement of CPV2c in a severe outbreak of typical haemorrhagic gastroenteritis in a susceptible litter of pups in Scandinavia.

## Background

Canine parvovirus 2 (CPV2) emerged during the late 1970s, probably as a result of mutation of feline parvovirus or a related parvovirus of wild carnivores [1]. CPV2 showed relatively rapid genetic evolution and within only a few years the first antigenic variants, CPV2a and CPV2b, appeared [2,3]. These variants replaced the original type 2 virus in the field, and from the mid-1980s until 2000 the presence of CPV2a and/or CPV2b isolates was widespread in all countries, although the relative proportions of these two types varied from country to country [1-8]. In 2000, a new variant, now referred to as CPV2c, was identified in Italy [9]. This new variant has since been identified in many other countries worldwide but the earliest evidence of its presence (based on retrospective testing) is from 1996 in Germany [10]. Despite CPV2c having now become established in geographically distinct countries worldwide, and in some countries (e.g. Italy, Argentina, Uruguay) having become the most frequently isolated variant [10-13], there are still countries where it has apparently not yet established. In the UK, for instance, apart from the discovery of a single isolate from Scotland, recent analysis of isolates from a large cross-sectional sample of UK dogs over a two year period with severe diarrhoea revealed the presence of 2a and 2b but not 2c [14]. Up until now there have been no isolates of CPV2c reported from Sweden, or any other Scandinavian country. The case reported here is of a severe outbreak of haemorrhagic gastroenteritis in a recently vaccinated litter of Swedish puppies where parvovirus was isolated. Following virus isolation and typing in order to confirm whether this was vaccine strain it was found to be a field strain of type 2c. This is believed to be the first report of the confirmation of the identification of CPV2c from a clinical case in Scandinavia.

## Case presentation

### History and clinical signs

A two year old, mixed breed, collie x berner sennen bitch, with an unknown vaccination status was taken to an animal hospital after a few days of vomiting and anorexia. The bitch was thin and in poor condition, with a brownish, malodourous discharge from the vulva. Pregnancy was confirmed with x-ray and the stage of gestation was estimated to be at least day 61 with at least 13 puppies. Abdominal ultrasound showed that at least two pups had a low heart rate (120–150). As a consequence of the poor condition of the bitch, and the deteriorating condition of the puppies, it was decided to perform a caesarean section followed by ovariohysterectomy and 14 live puppies were delivered in this way.

Post-operatively, the bitch was tired and unwilling to allow the puppies to feed. The litter was therefore hand-reared from birth using formula milk. (Babydog Milk - Royal Canin). The bitch and puppies went home later the same day, but the bitch was re-hospitalised the following day with fever (41°C) and signs of vomiting and diarrhoea but no signs of bleeding. On examination she was found to have pale mucosae, an elevated heart rate (150 beats/minute), a weak pulse and low blood pressure. She was admitted to the hospital intensive care unit, and treated with antibiotics, aggressive intravenous fluid therapy and plasma, but continued to deteriorate. Abdominal ultrasound showed that several sections of the intestine were corrugated and that the gastric wall was moderately hypertrophic with free abdominal fluid. Despite intensive treatment for some hours her condition continued to worsen and so later that day she was euthanized.

The puppies were placed in a foster home and bottle-fed with formula milk. As the bitch had not suckled the puppies they had never received colostrum. After the bottle-feeding period, the puppies were given puppy food (Science Plan Puppy: All breeds - Hills). Two of the 14 puppies had died at some point during their time in the foster home but unfortunately no further details are available concerning the age of these pups or what clinical signs were seen prior to death. The 12 remaining puppies were vaccinated at eight weeks of age with a multivalent canine vaccine (Nobivac DHPPi – MSD Animal Health) containing live attenuated strains of canine distemper virus, canine adenovirus 2, canine parvovirus 2 and canine parainfluenza virus. They were transferred to their new homes two days later.

According to information from the foster home, all 12 puppies became ill with signs of gastroenteritis within five days of being rehomed. Eight of the puppies were admitted for care at the animal hospital. The four remaining puppies had milder signs and were treated symptomatically at home or by their local veterinarian. No further information is available concerning these puppies.

For the eight hospitalized puppies, the onset of clinical signs was between four and seven days after vaccination. Four of these puppies were admitted six days after vaccination and four were admitted seven to ten days after vaccination. All the puppies showed a similar clinical picture; they were mildly to moderately dehydrated with dry mucous membranes, normal heart and lung sounds and signs of mild to moderate pain on abdominal palpation. At time of admission their body temperatures varied between 38.1-39.4°C and three puppies subsequently showed pyrexia with temperatures of 39.7-40.5°C. All puppies were vomiting a watery, sometimes haemorrhagic, fluid and passing a voluminous watery haemorrhagic mucoid diarrhoea several times a day. In addition some of the puppies had ascarids in the vomitus and diarrhoea.

All the puppies were given intravenous fluid therapy with lactated Ringer’s solution and Ringer’s with glucose. Seven of them were also given plasma infusions. They were also all given intravenous antibiotic therapy (ampicillin which in three cases were changed to cefuroxime). Five puppies were followed on leukogram and all five showed marked leucopenia and neutropenia, which had normalized before discharge. All puppies survived and hospitalization times ranged from 4–16 days (median 6 days).

### Virus identification and isolation

A faecal sample taken by the referring veterinarian from one puppy one day after the onset of clinical signs tested positive for parvovirus (Witness CPV Canine Parvovirus Antigen Test Kit - Pfizer). At the animal hospital, faecal samples were collected from four puppies (4–5 days after the onset of clinical signs) and stored at +8°C for about three months prior to being sent for laboratory analysis. Three of the samples (1, 2 and 3) were of a liquid consistency and were diluted in phosphate buffered saline (PBS), vortexed and filtered through a 45 μ filter. The fourth sample (4) was of a hard consistency however, and was homogenized with phosphate buffered saline (PBS) prior to filtering. All samples were examined for CPV content by growth in tissue culture on canine A72 cells, and also by the polymerase chain reaction (PCR) using primers specifically designed to enable differentiation of the CPV vaccine strain that is used in DHPPi (strain 154att), and all other vaccines and field CPV field viruses.

The filtered samples were inoculated onto A72 cells at the time of cell seeding and incubated at 37°C for four days. Cultures were examined daily for evidence of a developing cytopathic effect (cpe) and also by a haemagglutination assay (HA) using porcine erythrocytes. Although an obvious cpe was not observed, one isolate (4) was HA positive indicating the presence of CPV. As the cultures had reached cell confluency, a second viral pass was made by trypsinisation of the cell monlayer and splitting the culture into new flasks. On the second pass another sample (3) was HA positive and showed a cpe typical of CPV. All pass 2 cultures were harvested and titrated on A72 cells on 96 well plates for confirmation of CPV isolation. After incubation for 4 days at 37°C the cells were fixed with methanol and immunologically stained with a CPV specific monoclonal primary antibody and an anti-mouse conjugated (Fluorescein-isothiocyanate) secondary antibody. The two samples (3 and 4) gave positive immuno-staining of infected cells confirming the isolation of CPV, whereas the two HA negative samples (1 and 2) were also negative for isolation by immuno-staining.

### Virus typing

The sample filtrates were boiled for five minutes, cooled on ice, and 200 μl sample taken for purification and isolation of DNA template on a Qiagen QIA quick PCR clean up column according to the suppliers’ protocol. DNA was eluted from the column with 50 μl elution buffer and 5 μl used in a 50 μl Qiagen HotStarTaq master mix PCR reaction. The four sample templates were used in three different PCR reactions that are used routinely for the characterisation of clinical samples submitted where CPV infection is suspected. The PCRs have been designed to firstly detect the presence of CPV and secondly to differentiate the vaccine strain used in DHPPi, namely 154att, and all other CPVs.

### PCR 1: detection of vaccine virus

The specific detection of the 154att vaccine virus was made using primers located upstream of the VP2 gene; a forward primer specific to the vaccine sequence and a universal conserved reverse primer. In an extensive survey (unpublished), the vaccine specific sequence was not detected in over one hundred field viruses (types 2, 2a, 2b and 2c) collected from dogs with clinical parvovirus disease, many CPV vaccines or published non-structural sequences.

### PCR 2: detection of non-154att vaccine virus

The detection of non-154att CPVs was made by replacing the 154att specific primer in PCR 1 with a conserved forward primer.

### PCR 3: detection of all CPV types

Detection of all CPV types was made using conserved forward and reverse primers that amplified a 461 bp product that encapsidates the 426 amino acid residue.

PCR1, 2 and 3 were performed as follows: an initial denaturation step at 95°C for 15 mins, followed by 5 cycles of 94°C for 45 s, 45°C for 45 s and 72°C for 1 min, then 28 cycles of 94°C for 45 s, 50°C for 45 s and 72°C for 1 min, and a final extension step at 72°C for 10 mins.

In our experience the diagnostic PCRs described are as sensitive as viral isolation in tissue culture for the detection of CPV, and also have the advantage of a rapid diagnosis of disease.

Following PCR the samples were electrophoresed on a 0.8% agarose submarine gel containing ethidium bromide (Tris Borate EDTA running buffer) and an image recorded on a Biorad Gel Doc. All samples were positive for CPV and field virus; positive in PCR 2 and 3 respectively. However all samples were negative for the 154att strain in PCR 1. As the 154att vaccine was not detected this indicated that the CPV was most probably a field virus. Although the PCRs described enable the detection of CPV and the differentiation of vaccine (154att strain) from all other CPVs, they do not enable determination of the CPV antigenic type.

To determine the antigenic type of the isolates, the PCR 3 product was purified using a Qiagen QIA quick column and sequence determined on both strands on a ABI 3170XL instrument. Sequence alignments were made with reference CPV type 2, 2a, 2b and 2c sequences using Clone Manager version 11. These alignments showed that all four samples had a Glu at position 426 of the capsid and were therefore typed as CPV2c. Alignment of the Swedish VP2 with three Italian CPV2c isolates (Accession nos. FJ222821, FJ005233, GU362935) and one German CPV2c isolate (FJ005198) showed that the amino acid sequences were the same. However, the Swedish VP2 had a silent change at residue 364 (Alanine) with a GCA in place of GCG.

To confirm the CPV 2c typing of the isolates, preparations of the original faecal sample material were remade and DNA purified as described. The whole VP2 capsid gene was amplified in PCR4 using primers EF (forward) and JS2 (reverse) in a 50 μl reaction using Qiagen LongRange according to manufacturer’s instructions. Following an initial denaturation step at 93°C for 3 mins, amplification was carried out following 35 cycles of 93°C for 15 s, 52°C for 30s and 68°C for 2 mins. Sequence was determined on both strands confirming that all the isolates were identical and indeed CPV2C.

To address the possibility of cross contamination, all work was carried out to ‘good laboratory practice’ and non-infected cell controls and no DNA reagent controls were included in virus isolation in tissue culture and PCR assays respectively.

Primer oligonucleotides used for amplification of capsid VP2 and sequence determination are detailed in Table 1.

**Table 1:** Primer oligonucleotides used for amplification of capsid VP2 and sequence determination

**PCR**	**Primer name**	**5′ – 3′ primer sequence**	**Position in 154att**	**Fragment size**	**Reference**
3	NG5	ATTCCTGTTTTACCTCCAATTGGATCTGTT	R 4090	461	MSD AH
	NG6	AAATAGAGCATTGGGCTTACCACCATTTTT	F 3629		MSD AH
4	EF	GCCGGTGCAGGACAAGTA	F2748	2051	[15]
	JS2R	CCACCCACACCATAACAACA	R 4799		[16]

## Conclusions

This case report raises a number of interesting issues. First, although a full diagnostic screen for other pathogens was not undertaken, the presence of a field isolate of CPV in faecal samples taken from a number of the pups which were showing signs of severe haemorrhagic gastroenteritis at the time would normally be considered sufficient evidence to conclude that this was indeed an outbreak of parvoviral enteritis [17,18].

The source of the infection is not known but from the fact that the timing of onset of clinical disease was 2–5 days after the pups had been transferred from the foster home to their various new homes this makes it certain that the pups must have become infected at the foster home, and probably just before leaving bearing in mind the usual incubation period of 3–5 days for canine parvovirus [19]. It is not known whether there was a sick dog at the foster home at around this time, or if someone at the home had either been in contact with a clinical case or otherwise picked up infected material but these would be the usual explanations for the source of infection.

Although the vaccination history of the bitch was unknown, the pups had been vaccinated with a parvovirus-containing multivalent vaccine (Nobivac DHPPi – MSD Animal Health) a few days before the onset of signs in the majority of pups. There has been some debate about whether vaccination – especially with vaccines based on the original ‘type 2’ virus – will protect as effectively against the more recent subtypes, especially the 2b and 2c variants, although a number of studies have shown evidence of cross-protection in this respect [20-23]. In this case however it is clear from the timing of the onset of clinical signs that the pups were already incubating infection and that also the vaccine would probably not have had a chance to stimulate a protective immunity (the manufacturers claim a 7–10 day onset of immunity post-vaccination). Therefore in this case vaccination could not have been expected to be effective.

As regards vaccine efficacy, another caveat when vaccinating pups younger than around 3 months of age is the possibility of interference from maternally-derived antibody (MDA) which is transferred from the bitch mainly via the colostrum shortly after birth. Following a normal parturition and post-parturient period, puppies would normally be expected to have acquired reasonable levels of MDA - assuming that the bitch had a reasonable level of immunity to parvovirus - and should be protected against disease for the first few weeks of life [24]. In this case however, with the history of no intake of colostrum, it can reasonably be assumed that the pups would have had minimal, if any, levels of MDA and should probably have been vaccinated much earlier than 8 weeks of age. In this respect in cases where MDA is known to be low, vaccination of the pups from at least six weeks and possibly four weeks of age has been suggested [25-27]. Although the use of many canine parvovirus vaccines at such a young age is strictly ‘off label’ there are some so-called ‘puppy vaccines’ which have been shown to be safe at these ages and can therefore be used in this way in line with the manufacturer’s recommendations.

Although the CPV isolate from the pups was confirmed as a CPV type 2c, and therefore was not the vaccine strain, one common concern of many owners and veterinarians in cases where recently-vaccinated pups show signs of typical clinical disease would be whether the live vaccine virus could have reverted to virulence and be responsible for the outbreak of disease [28]. In this case, this was indeed the main reason that the virus was sent for further identification following the initial confirmation of the presence of CPV in the faeces using an in-clinic test kit. Quick test kits can detect the presence of parvovirus but will not be able to distinguish the type involved. In order to confirm whether the isolate is a field strain or vaccine strain it is often sufficient to type the virus since the original ‘type 2’ virus is no longer present in the field [2] and many vaccines (as was the case here) are based on a type 2 strain. In such cases the identification of either type 2a, 2b or 2c would be sufficient to confirm a field infection. In this case the manufacturers were initially able to use specific PCR probes to differentiate between the unique genetic fingerprint of the vaccine strain and all other CPV strains, thus confirming that the isolate was not vaccine strain. Follow-up typing was then used to determine that the isolate was in fact a type 2c.

CPV2c has been reported as the most recent type of CPV to have evolved and was first discovered in Italy in 2000 [9]. Subsequently this type has been identified in many other countries worldwide [11,29] but to date there have been no reports of its presence in Sweden, or any other Scandinavian countries. This case report therefore represents the first published evidence of the involvement of CPV2c in a severe outbreak of typical haemorrhagic gastroenteritis in a susceptible litter of pups in Scandinavia.

## Competing interests

AG, NG, JP and DS are employees of MSD Animal Health, the manufacturer of the Nobivac vaccines used in the puppies which are the subject of this case report. MSD Animal Health paid the publication fee for this article.

## Authors’ contributions

CV was responsible for providing details of the case history and clinical signs from practice records. NS and JP were responsible for the virus isolation, identification and typing. All authors have been involved in drafting the manuscript. All authors have given final approval of the manuscript.
